# The Impact of Vitamin B12 Supplementation on Clinical Outcomes in Patients With Diabetic Neuropathy: A Meta-Analysis of Randomized Controlled Trials

**DOI:** 10.7759/cureus.31783

**Published:** 2022-11-22

**Authors:** Jithin Karedath, Saima Batool, Abia Arshad, Sumon Khalique, Sooraj Raja, Bihari Lal, Venkata Anirudh Chunchu, Shamsha Hirani

**Affiliations:** 1 Internal Medicine, James Cook University Hospital, Middlesbrough, GBR; 2 Internal Medicine, Hameed Latif Hospital, Lahore, PAK; 3 Medicine, Dow International Medical College, Karachi, PAK; 4 Medicine, Shafay Hospital, Hyderabad, PAK; 5 Internal Medicine, Jinnah Sindh Medical University, Karachi, PAK; 6 Medicine, Chandka Medical College, Shikarpur, PAK; 7 Medicine, Avalon University School of Medicine, Willemstad, CUW; 8 Cardiology, Baqai Hospital, Karachi, PAK

**Keywords:** meta-analysis, neuropathic symptoms, diabetes, vitamin b12, diabetic neuropathy

## Abstract

Diabetic neuropathy (DN) is one of the most prevalent and expensive microvascular consequences of diabetes mellitus (DM), which is noteworthy given that it is frequently both underdiagnosed and undertreated in daily clinical practice. The aim of the current article was to review the efficiency of vitamin B12 supplementation in isolation or in combination therapy for the treatment of diabetic peripheral neuropathy. This meta-analysis was designed according to the Preferred Reporting Items for Systematic Reviews and Meta-Analyses (PRISMA). A systematic electronic search was performed in PubMed and Cochrane Library to identify randomized controlled trials (RCTs) assessing the impact of vitamin B12 outcomes in patients with diabetic neuropathy without putting restrictions on the year of publication. A combination of the following keywords was used: “diabetic neuropathy,” “vitamin B12,” and “outcomes.” The primary outcomes assessed in the current meta-analysis included neuropathic symptoms and vibration perception threshold (VPT). Secondary outcomes included a change in pain score from baseline, total cholesterol (mg/dL), high-density lipoprotein (HDL), and low-density lipoprotein (LDL). A total of six articles were selected to be included in the current meta-analysis. Patients receiving vitamin B12 showed a greater reduction of mean neuropathic symptoms (standardized mean difference (SMD): -0.39, 95% confidence interval (CI): -0.73, -0.05, p-value: 0.03) and pain score (SMD: -3.60, 95% CI: -4.68, -1.43, p-value<0.001) compared to the control group. No significant effect of vitamin B12 was found on VPT (mean difference (MD): -4.80, 95% CI: -11.03, 1.42, p-value: 0.13), change in HDL (MD: 0.14, 95% CI: -2.37, 2.65, p-value: 0.91), LDL (MD: 2.59, 95% CI: -5.94, 11.12, p-value: 0.55), and total cholesterol (MD: -2.72, 95% CI: -11.52, 6.08, p-value: 0.54). The current meta-analysis found that vitamin B12 can improve neuropathic symptoms and reduce pain in patients with diabetic neuropathy. However, the current study did not report any significant difference between patients who received vitamin B12 and placebo in terms of HDL, LDL, and total cholesterol.

## Introduction and background

Diabetic neuropathy (DN) is one of the most prevalent and expensive microvascular consequences of diabetes mellitus (DM), which is noteworthy given that it is frequently both underdiagnosed and undertreated in daily clinical practice [1]. Nearly 59% of patients with type 1 diabetes mellitus and 50% of patients with type 2 diabetes mellitus develop diabetic peripheral neuropathy [2]. Some diabetic peripheral neuropathy patients may have excruciatingly painful symptoms, but people with more severe neuropathic deficits might not experience any symptoms [3]. More than 30% of patients develop peripheral diabetic neuropathy with pain and symptoms such as needles, pins, burning and hot or cold sensations, dead feeling and numbness in the contact, and leg and foot pain [4,5]. These can have a significant impact on the quality of life [4].

Lack of methylcobalamin, which is caused by a deficiency in vitamin B12, has been linked to substantial neurological disease, particularly peripheral neuropathy [6]. It also heralds the beginning of diabetic neuropathy. Vitamin B12 insufficiency in diabetic peripheral neuropathic patients may result from the utilization of antidiabetic medications such as metformin [7]. Along with any kind of anti-glycemic therapy pointing at strict glycemic control, vitamin B12 has been the commonly used supplement because vitamin deficiency of vitamin B12 is common in type 2 diabetic patients. Besides this, a deficiency of vitamin B12 is more likely to cause neurological symptoms such as painful neuropathy and autonomic neuropathy [1-8]. Vitamin B12 is regarded as an analgesic medication in several nations. In the descending inhibitory nociceptive system, it has been hypothesized that vitamin B12 may improve the availability and potency of noradrenaline and 5-hydroxytryptamine [9].

The American Diabetes Association (ADA) suggests that patients with diabetic neuropathy on metformin therapy have their vitamin B12 levels periodically checked once a year [10]. The usage of metformin has been primarily attributed to the deficiency of vitamin B12 in type 2 diabetes mellitus. Since more than 40 years ago, there has been evidence linking metformin to a deficiency of vitamin B12. Several interventional investigations, observational studies, and meta-analyses have supported this association [11,12].

As far as our knowledge is concerned, no previous meta-analysis has been conducted to determine the efficiency of vitamin B12 in patients with diabetic neuropathy. Clinical trials assessing the impact of vitamin B12 enrolled a small sample size. Thus, the current meta-analysis was conducted to assess the impact of vitamin B12 in patients with diabetic neuropathy using a large sample size. The current article aimed to review the efficiency of vitamin B12 supplementation in isolation or combination therapy for the treatment of diabetic peripheral neuropathy.

## Review

Methodology

This meta-analysis was designed according to the Preferred Reporting Items for Systematic Reviews and Meta-Analyses (PRISMA).

Search Strategy and Study Selection

A systematic electronic search was performed in PubMed and Cochrane Library to identify randomized controlled trials (RCTs) assessing the impact of vitamin B12 outcomes in patients with diabetic neuropathy without putting restrictions on the year of publication. A combination of the following keywords was used: “diabetic neuropathy,” “vitamin B12,” and “outcomes.” The title and abstract were screened for relevancy, and the full text of relevant articles was retrieved to assess for eligibility. The reference lists of all eligible studies were manually screened to search for additional studies. The selection of studies was done by two authors independently.

Studies that assessed any type of vitamin B12 therapy, including coenzyme forms of vitamin B12, in either injection or oral form were eligible to be included in the current meta-analysis. Studies involving combination therapy, for example, if vitamin B12 or its coenzyme forms was one of the treatment agents along with other therapies, were also included in this meta-analysis. Studies involving diabetic neuropathy were part of this meta-analysis. Peripheral large or small fiber neuropathy that causes autonomic or somatic sensory problems is known as diabetic neuropathy. Studies other than RCTs were not included in the current meta-analysis. Besides this, studies that did not report desired outcomes were also not included in the current meta-analysis. Studies published in a language other than English were also not included.

Outcomes

The primary outcomes assessed in the current meta-analysis included neuropathic symptoms and vibration perception threshold (VPT). Neuropathic symptoms were assessed using the Michigan Neuropathy Screening Instrument Questionnaire (MNSIQ) and Neuropathy Symptom Score (NSS). VPT is a simple way of detecting large fiber dysfunction, thus identifying individuals with diabetes at risk of ulceration. Secondary outcomes included a change in pain score from baseline, total cholesterol (mg/dL), high-density lipoprotein (HDL), and low-density lipoprotein (LDL).

Data Extraction

Data were extracted from included studies using predesigned data extraction forms. Data from the included studies were extracted by one author and double-checked and entered into Review Manager (RevMan) software (The Nordic Cochrane Centre, The Cochrane Collaboration, Copenhagen, Denmark) by the second author. Data extracted included author name, publication year, groups, sample size, follow-up period, mean age, and gender.

Risk of Assessment

The risk of bias in the current meta-analysis was assessed using the Cochrane risk of bias assessment. The risk of bias was assessed by two authors independently. The risk of bias tool covers six domains of bias including “selection bias,” “performance bias,” “detection bias,” “attrition bias,” “reporting bias,” and “other bias.” Any disagreement between the two authors was resolved through discussion.

Statistical Analysis

Review Manager (RevMan) version 5.4.0 (The Nordic Cochrane Centre, The Cochrane Collaboration, Copenhagen, Denmark) was used to perform a meta-analysis. A standardized mean difference (SMD) along with a 95% confidence interval (95% CI) was reported to estimate the effect of vitamin B12 on the improvement of neuropathic symptoms, VPT, and pain score. To estimate the effect of vitamin B12 on HDL, LDL, and total cholesterol, the mean difference (MD) and 95% CI were computed. Heterogeneity among the study results was calculated by calculating I-square statistics. In the case of an I-square value of <50%, a fixed-effect model was used.

Results

**Figure 1:**
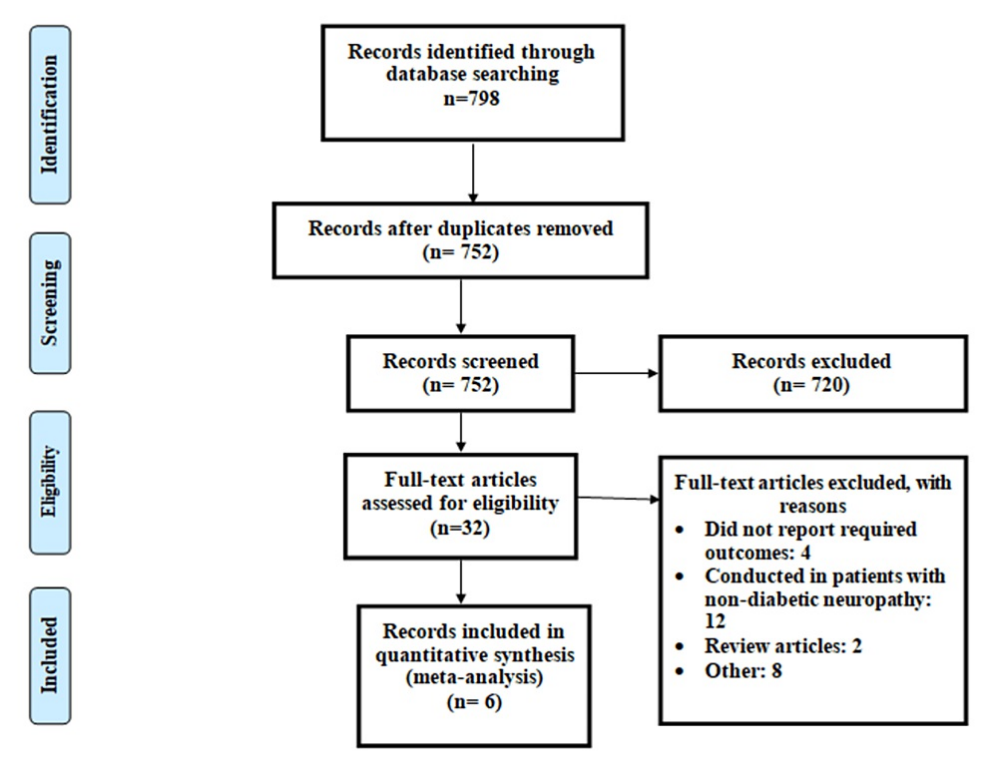
PRISMA flowchart of the selection of studies PRISMA: Preferred Reporting Items for Systematic Reviews and Meta-Analyses

The process of study selection is summarized in Figure 1. Out of 798 articles identified through online searching, title and abstract screening of 752 articles was done to assess for eligibility. Out of 752 articles, 32 articles were retrieved for full-text screening. Six articles were selected to be included in the current meta-analysis [13-18]. Table 1 shows the characteristics of the included studies.

**Table 1:** Characteristics of the included studies SOD: superoxide dismutase; ALA: alpha lipoic acid; ALC: acetyl-carnitine

Author name	Publication year	Groups	Intervention	Sample size	Follow-up (months)	Mean age (years)	Males (number (%))
Didangelos et al. [13]	2020	Vitamin B	SOD, ALA, vitamin B12, and ALC	43	18	64.23	44 (51.76)
		Placebo		42			
Didangelos et al. [14]	2021	Vitamin B	Vitamin B12	44	12	62.85	48 (53.33)
		Placebo		46			
Farvid et al. [15]	2011	Vitamin B	Minerals, vitamin B1, vitamin B2, vitamin B6, biotin, vitamin B12, and folic acid	22	4	53.55	23 (52.27)
		Placebo		22			
Fonseca et al. [16]	2013	Vitamin B	Methylfolate calcium, methylcobalamin, and pyridoxal-5-phosphate	106	6	62.62	148 (69.16)
		Placebo		108			
Li et al. [17]	2016	Vitamin B	Vitamin B12	115	6	57.78	122 (52.59)
		ALC		117			
Stracke et al. [18]	1996	Vitamin B	Vitamin B1, vitamin B6, and vitamin 12	11	3	59	14 (58.33)
		Placebo		13			

**Figure 2:**
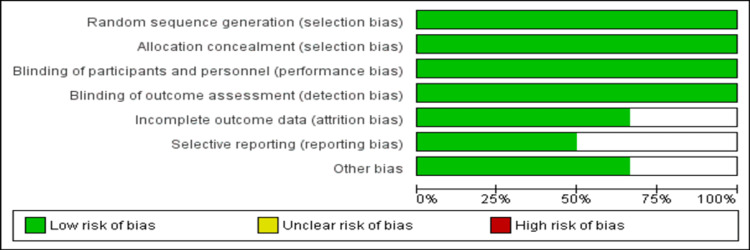
Assessment of risk of bias

The pooled mean age of patients was 60.45 years. The majority of patients were males in all the included studies. In four RCTs, vitamin B12 was given in combination with other vitamins and minerals [13,15,16,18], while in two studies, vitamin B12 was given alone [12,17]. Figure 2 shows the overall risk of bias assessment. The overall quality of the study was high.

**Figure 3:**
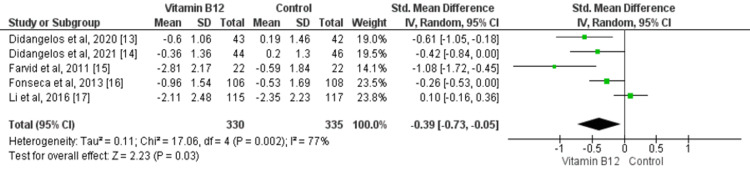
Forest plot of the association between vitamin B12 supplementation and neuropathic symptoms Sources: [13-17] SD: standard deviation; 95% CI: 95% confidence interval

A meta-analysis of the effect of vitamin B12 on neuropathic symptoms is shown in Figure 3. Overall, five studies assessed the effect of vitamin B12 on neuropathic symptoms [13-17]. Patients who received vitamin B12 showed a greater reduction of mean neuropathic symptoms compared to the control group (SMD: -0.39, 95% CI: -0.73, -0.05, p-value: 0.03). Significant heterogeneity was found among the study results (I-square: 77%, p-value: 0.02).

**Figure 4:**
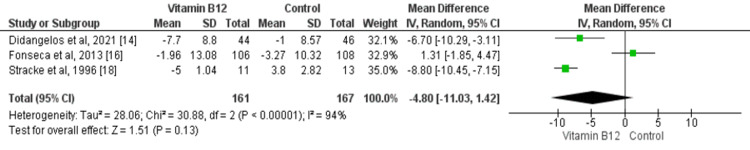
Forest plot of the association between vitamin B12 supplementation and VPT Sources: [14,16,18] VPT: vibration perception threshold; SD: standard deviation; 95% CI: 95% confidence interval

Three studies assessed the impact of vitamin B12 supplementation on VPT [14,16,18]. The random effect model showed that no significant effect of vitamin B12 was found on VPT (mean difference: -4.80, 95% CI: -11.03, 1.42, p-value: 0.13) as shown in Figure 4. Significant heterogeneity was found among the study results (I-square: 94%, p-value: 0.001).

**Figure 5:**
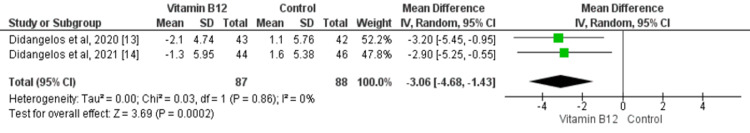
Forest plot of the association between vitamin B12 supplementation and pain score Sources: [13,14] SD: standard deviation; 95% CI: 95% confidence interval

Two studies compared the impact of vitamin B12 on the change in pain score between patients who received vitamin B12 and patients who received placebo [13,14]. Patients who received vitamin B12 experienced greater pain reduction scores compared to patients who received placebo (SMD: -3.60, 95% CI: -4.68, -1.43, p-value<0.001) as shown in Figure 5. No significant heterogeneity was found among the study results (I-square: 0%, p-value: 0.86).

**Figure 6:**
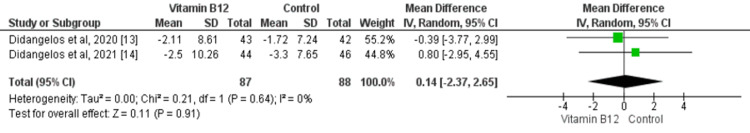
Forest plot of the association between vitamin B12 supplementation and HDL change Sources: [13,14] HDL: high-density lipoprotein; SD: standard deviation; 95% CI: 95% confidence interval

**Figure 7:**
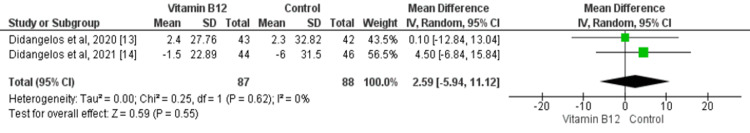
Forest plot of the association between vitamin B12 supplementation and LDL change Sources: [13,14] LDL: low-density lipoprotein; SD: standard deviation; 95% CI: 95% confidence interval

**Figure 8:**
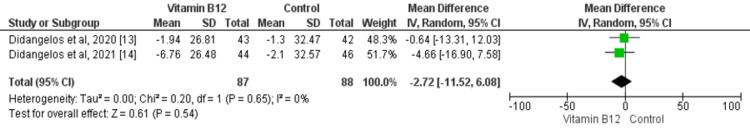
Forest plot of the association between vitamin B12 supplementation and total cholesterol change Sources: [13,14] SD: standard deviation; 95% CI: 95% confidence interval

Two studies assessed the impact of vitamin B12 on HDL, LDL, and total cholesterol in patients with diabetic neuropathy. No significant differences were found between two groups in terms of change in HDL (mean difference: 0.14, 95% CI: -2.37, 2.65, p-value: 0.91), LDL (mean difference: 2.59, 95% CI: -5.94, 11.12, p-value: 0.55), and total cholesterol (mean difference: -2.72, 95% CI: -11.52, 6.08, p-value: 0.54) as shown in Figure 6, Figure 7, and Figure 8, respectively. No significant heterogeneity was found among the study results in any of the outcomes.

Discussion

The current meta-analysis provided an updated overview of the impact of vitamin B12 on clinical outcomes in patients with diabetic neuropathy. The current meta-analysis showed that patients who received B12 alone or in combination with other drugs experience a greater reduction of mean neuropathic symptoms compared with placebo. In addition, they experienced greater pain reduction scores compared to patients who received placebo.

Diabetic neuropathy is a serious complication of type 2 diabetes mellitus, and it can lead to devastating symptoms such as unbearable and unremitting pain and can have severe life-threatening outcomes including the “diabetic foot” [19]. Strict glycemic control is now the only known non-symptomatic treatment for diabetic neuropathy; however, its effectiveness in treating diabetic peripheral neuropathy is quite limited, and it must be maintained for years [20]. Thus, there is an urgent requirement for an efficient drug therapy that would act casually through the modification of the diabetic neuropathy pathophysiology. Among other supplements and drugs such as superoxide dismutase, folate, carnitine, and alpha lipoic acid, vitamin B12 has been the most often used drug [21]. This is due to the fact that deficiency of vitamin B12 is commonly found in individuals with type 2 diabetes mellitus, and deficiency of vitamin B12 may lead to several neurological disorders accelerating diabetic neuropathy [22].

The current meta-analysis supported the point that vitamin B12 resulted in an improvement in pain. A systematic review conducted by Li et al. showed similar effects [23]. These findings can assure the analgesic action of vitamin B12 that is potentially arbitrated by a rise in the effectiveness and availability of 5-hydroxytryptamine and noradrenaline [24]. Vitamin B12 occurs in different forms called cobalamins. The main form of cobalamin utilized in vitamin supplements is cyanocobalamin, while methylcobalamin is a coenzyme form that is a crucial cofactor for the function of vitamin B12-dependent methyltransferases [25].

Among five studies that assessed the improvement of neuropathic symptoms, a study conducted by Li et al. [17] found no significant difference in relation to neuropathic symptom improvements. This study used acetyl-L-carnitine (ALC) as a control group and reported that in both groups (vitamin B12 and ALC), neuropathic symptom score was significantly lower at 24 weeks as compared to the baseline [18], and no significant difference was observed between the two groups. Another study that used vitamin B12 alone reported that vitamin B12 significantly reduced neuropathic symptoms [14].

The current meta-analysis is associated with certain limitations. Firstly, less number of included studies considered homocysteine levels and methylmalonic acid as potential confounders for the response of vitamin B12 therapy. Secondly, due to the lack of individual-level data, we were not able to perform subgroup analyses of age, gender, and comorbidities. Thirdly, among all the included studies, two used vitamin B12 alone, while six studies involved combination therapy in which one of the components was vitamin B12. Besides this, there was a great variation in these studies in terms of mode of administration, molecular form, dose, duration of follow-up, and baseline blood levels. Lastly, only six studies were included in the current meta-analysis, and the sample size is relatively small in all the included studies, which impacts the power of the study findings. However, this is the first meta-analysis conducted on the impact of vitamin B12 on clinical outcomes in patients with diabetic neuropathy that can give some idea of the utilization of vitamin B12 in patients with diabetic neuropathy. In the future, more prospective randomized controlled trials are required to guide clinical practice.

## Conclusions

The current meta-analysis was conducted with the aim to assess the impact of vitamin B12 alone or in combination with other treatments in patients with diabetic neuropathy. The current meta-analysis found that vitamin B12 can improve neuropathic symptoms and reduce pain in patients with diabetic neuropathy. However, the current study did not report any significant difference between patients who received vitamin B12 and placebo in terms of HDL, LDL, and total cholesterol.
